# Dr. Morand on Medicine and Surgery

**Published:** 1847-04

**Authors:** 


### Art. III.

*Mémoires et Observations Cliniques de Médecine et de Chirurgie.* Par L. Morand, m.d., &c.—*Tours,* 1845.

*Clinical Essays and Observations in Medicine and Surgery.* By L. Morand, m.d., &c.—*Tours,* 1845. 8vo, pp. 258.

This volume consists of memoirs on scrofulous ophthalmia, on hernia, on diphtheritis, &c., together with a series of reports of the more interesting medical and surgical cases that have occurred in the practice of M. Morand, a surgeon of Tours. To these is added a description of two new instruments invented by the author—an obstetrical forceps and a gum-lancet. Although the majority of the essays contained in this work are neither, in point of novelty or of practical importance, of a higher order than the average run of "Original communications" to journals—in which several of them have already appeared—yet as some of them contain facts that may be interesting to our readers, we will give a succinct analysis of the principal papers, taking them in the order in which they are presented to us by M. Morand.

*Scrofulous ophthalmia.* The first essay in situation as well as importance is on the coincidence of scrofulous ophthalmia with inflammation of the pituitary membrane, and on the necessity of treating the affection of the nose before undertaking the cure of the disease of the conjunctiva. This memoir, it appears, was laid before the Royal Academy of Medicine of Paris, and was favorably reported upon by Velpeau, who was appointed to examine into its merits. It consists of an account of an epidemic ophthalmia that broke out in 1841, amongst the children inhabiting some model-farms at Mettray. In the course of this epidemic, M. Morand's attention was especially directed to the state of the pituitary membrane, which he uniformly found to be in a congested or even inflammatory condition; and so intimately was this state of the mucous membrane of the nose connected with the conjunctivitis, that he could foretell the probable supervention of the latter disease on finding the nasal cavities reddened or irritated, and the Schneiderian membrane congested or inflamed. Before being affected with ophthalmia, many children likewise suffered from the ordinary symptoms of coryza. The continued observation of these facts led M. Morand to the conclusion that this condition of the nostrils preceded the inflammation of the conjunctiva, of which it was, as it were, the starting-point, and occasioned by its persistence the numerous relapses that his patients suffered from.

"It results, from my observations," says M. Morand, "that in scrofulous ophthalmia the olfactory membrane participates with the conjunctiva in the inflammation that is set up; that it is especially about the turbinate bones, and in the anfractuosities of the nasal fossæ that the inflammatory action resides, and that this shows itself in the form of an œdematous engorgement precisely similar to what is observed in the eyelids. The more I study this disease the more convinced am I that it is so. A little attention suffices to show that the redness and tumefactions of the pituitary membrane almost always precede or accompany that of the conjunctiva. This can be more positively determined by means of the speculum auris. On examining attentively the interior of the nasal fossæ, one cannot fail to observe that the redness and swelling of the nostrils, and even of the upper part of the lip, that are so commonly observed in persons of a scrofulous habit, are merely an evidence of the inflammatory action going on in that membrane. It is by proceeding in this way that we can best appreciate the degree and extent of this inflammatory action, the extension of which to the palpebral and ocular mucous surfaces is often very rapid; sometimes, however, it remains for a long time stationary, without showing any disposition to extend." (p. 3-4.)

From the repeated observation of the coexistence of the inflammations of the Schneiderian membrane and of the conjunctiva, M. Morand was led to adopt a new mode of treatment, which appears to have been pretty successful. Instead of applying his remedies to the eyelids or eyes, he attacked the disease existing in the interior of the nose. Before making the observation that has just been referred to, M. Morand had tried a variety of general and local means; two oculists of eminence had been consulted, but without avail; the ophthalmia resisted most obstinately, and cases continued to occur with a frequency that caused considerable uneasiness to the directors of the farms at Mettray. From the time, however, that he determined to attack the disease in the nostrils, the inflammation of the eyes speedily subsided, and relapses were much less frequent than before. The plan adopted by M. Morand was to cauterize the interior of the nostril of the side corresponding to the affected eye with nitrate of silver, in the solid form or in solution, or as ointment, every morning and evening for the first week; after this once a day, and when the disease was declining, every second or third day. He enters a caution against cauterizing the aperture of the nose, and states that as soon as the caustic has been applied, the patient should make some deep inspirations through the nostrils, so as to spread the application over as large a surface as possible.

In an appendix to the memoir that we are now considering, M. Morand states that he has latterly had occasion to examine twenty-one patients with scrofulous inflammations of the conjunctiva, and that in nineteen of these cases he found the inflammation of the pituitary membrane to be more or less marked. He likewise observed that whenever there was an increased degree of irritation in the nasal fossæ, there was a marked exacerbation of the conjunctival inflammation, and frequently considerable tumefaction of the upper lip.

In order to determine the existence of the irritation of the pituitary membrane, it is by no means always necessary to look for redness of the nostrils and swelling of the upper lip; for, in the absence of these signs, it may easily be recognized by the constant flow of a more or less viscid mucus from the nostril that corresponds to the inflamed eye. It is also easy to ascertain the existence of this irritation by the introduction of the speculum nasi, or of a probe into the nasal fossæ, by which means any constriction or congestion may be readily detected. M. Morand has likewise found that the swelling of the upper lip, which frequently assumes an eczematous character, will very commonly yield to the application of the nitrate of silver, in one or other of the forms already mentioned, to the interior of the nostrils, when all other means have failed to relieve it.

It has long been known to surgeons that conjunctivitis is by no means an unfrequent complication of eczema of the upper lip and of the scalp, and the facts adduced by M. Morand in proof of the coexistence of inflammation of the ocular and nasal mucous membranes are interesting, as belonging to the same class with these; and although he may perhaps have overstated the frequency of this complication, yet the possibility of its occurrence should be kept in mind, and in all cases in which the ophthalmia is more than usually obstinate, it would be well not to overlook the condition of the nasal fossæ. With regard to the application of the caustic to the interior of the nostrils, instead of to the eye itself, we think, without recommending it, at all events, in every case, that it is a means that might be useful when the disease has evidently been primarily seated in the nose, or when, on account of the photophobia, it is difficult to separate the eyelids sufficiently to make the necessary local applications to the eyes: and there appears no reason to doubt that, if employed in conjunction with proper local and general treatment, it might materially tend to shorten the duration of attacks, and lessen the liability to relapse in cases of scrofulous conjunctivitis.

*Cataract.* The next subject that engages our author's attention is the question as to the propriety of operating for cataract when only one eye is affected. On this interesting point he adduces no arguments, but contents himself with relating the cases of two old men, on whom he operated by depression on one eye only, with perfect success; he does not mention, however, whether double vision followed the operation or not.

*Epistaxis.* We next come to what M. Morand is pleased to call a *new* means of arresting epistaxis, but which merely consists in plugging the nostrils with a piece of amadou rolled up in a piece of paper, instead of with lint or prepared sponge, as generally adopted.

*Leech-bites.* In order to arrest the hemorrhage from leech-bites, he recommends the application of a mixture of six parts of olive oil and two or three of yellow wax; this should be spread on a thin layer over the bleeding orifices, which must previously have been wiped dry.

There is nothing to detain us in the reports of two cases of *pericarditis* treated with blisters, colchicum, and antimonial wine; in two cases of *meningitis,* in which tartar emetic ointment was applied to the scalp with marked benefit; or in an instance of apparently *spontaneous gangrene* of the leg, in which amputation was had recourse to. Cases of this description, although interesting in themselves, are scarcely of sufficient importance to merit special notice here.

*Fistula.* The next case is intended to illustrate the advantages to be derived from the employment of stimulating injections for the cure of fistulæ, so situated that they cannot be laid open. It is that of a young woman who had suffered for nearly a twelvemonth from a fistulous opening in the right iliac fossa, consequent on an abscess in that region. After all the ordinary means of treatment had failed, it was resolved to try the effect of stimulating injections; for this purpose, a solution of the chloruret of soda was thrown in, with the best effect, the fistula being firmly closed in a fortnight.

*Hernia.* The next paper that we come to is a long memoir on the propriety of uniting by the first intention the wound that results from the operation for hernia. In this country it would, to say the least, be a work of supererogation for a surgeon to publish a memoir of from thirty to forty pages on the propriety of attempting, in certain cases, to heal the wound after an operation for hernia by the first intention, of bringing the edges together by a few strips of plaster, rather than dressing it from the bottom. In France, however, the case is different; the surgeon "leaves the wound open, and dresses it with a piece of perforated rag, spread with ointment and covered with pledgets of charpie and longitudinal compresses." (p. 79.) And it is amusing to see the hesitation with which M. Morand ventures to dissent from this practice. Although he admits that after union by the first intention, the cicatrix is small and linear, the rapidity of the cure is much increased, and the chances of those accidents that may complicate any wound diminished; whereas, by the after-treatment adopted in France, peritonitis, purulent absorption, and even tetanus are not unfrequent, and the resulting cicatrix is large, weak, and liable to excoriate. Yet he is far too patriotic not to come to the conclusion that "on reflecting on the advantages and disadvantages of these different plans, we cannot hesitate to follow that adopted by the French surgeons of the present day." (p. 79.)

We pass over without extract or comment, as presenting no feature of particular interest, the three reports that follow; one of a case of dothinenteritis, in which laudanum was administered with a view of allaying the delirium; the other a case of hooping-cough, complicated with an intermittent fever; and the third some cases illustrating the advantages of the "onguent Napolitain" in abscesses about the face; and proceed to the memoir on diphtheritis, the longest, and one of the most important essays in the volume.

*Diphtheritis.* M. Morand relates eleven cases of diphtheritis, which are interesting on account of their having been treated almost solely by cauterization, with a solution of the nitrate of silver, and apparently with a fair share of success, only two out of the eleven cases having proved fatal. He is a warm advocate of the direct application of the caustic to the seat of disease in these cases, and thinks, with his townsman Bretonneau, that when this means fails there is no resource left but in the performance of tracheotomy. He employs a solution of nitrate of silver, in the proportion of one part of the salt to three of water, and applies this freely three or four times a day. By these means the inflammation is modified, the fibrinous exudation is lessened, and the extension of the disease to the bronchi prevented. Should the disease continue in spite of this, there is nothing left for the surgeon to do but to open the trachea, and the proper moment for doing this is one of the most difficult questions that can present itself in practice. If done too early the patient's danger may be increased by the possibility of the supervention of bronchitis in consequence of the operation; if, on the other hand, it be delayed until the early stages of asphyxia have supervened, the lungs becoming engorged, the bronchi loaded with viscid mucus, hematosis effected with difficulty, and the prostration of the vital powers great, it will be next to impossible to prevent the mischief in the lungs from proceeding to a fatal termination—the patient dying asphyxiated. Under these circumstances an operation would be useless, and we think that we are only expressing the opinions of the best surgeons, when we say that it will seldom hold out a reasonable chance of success if it be performed after the lungs have become at all extensively engorged. In order to maintain the wound in the trachea sufficiently open, M. Morand has invented a dilator, which, although it may attain its object tolerably well, appears to us, from the representation given of it, to be far too cumbrous for ordinary use, and rather to be applicable to the purposes of a speculum auris or nasi, for which it seems likewise to have been intended.

*Incontinence of urine.* For the cure of incontinence of urine, M. Morand recommends the extract of belladonna administered in the form of pills; each of which contains a fifth of a grain of the extract. For children of from four to six years of age, one of these pills night and morning may suffice; if, however, at the end of a week no effects are produced, he recommends another at noon, and at the end of another week, a fourth at bedtime. Children of from eight to fifteen years of age may begin with three a day, and carry the dose up to six or even eight; and adults may take from twelve to fifteen. This treatment, whatever its general success may be, has the disadvantage of being somewhat tedious, from three to four months being the average time required for a cure to be effected; and even then the disease is liable to relapse, as we learn from M. Morand's own statement.

As the remaining two or three essays contain nothing of general interest, we must conclude our notice of this small work, which, though it possesses fewer claims to originality than its author appears to think, evidently emanates from the pen of an acute and intelligent practitioner.

